# Sintilimab-induced inflammatory myopathy in a patient with esophageal cancer: a case report

**DOI:** 10.3389/fimmu.2023.1253463

**Published:** 2023-10-18

**Authors:** Guo Hong, Haina Zhao, Yuxuan Yin, Hailin Shen, Zhaohao Zeng, Jianwei Yang, Lili Zhang

**Affiliations:** ^1^ Department of Neurology, Shenzhen People’s Hospital (The Second Clinical Medical College, Jinan University, The First Affiliated Hospital, Southern University of Science and Technology), Shenzhen, China; ^2^ Shenzhen Clinical Research Centre for Geriatrics, Shenzhen People’s Hospital, Shenzhen, China; ^3^ Department of Neurology, Nantong Branch of Renji Hospital, School of Medicine, Shanghai Jiao Tong University, Nantong, China; ^4^ Department of Neurology, Institutes of Brain Science, Jiangsu Subei People's Hospital affiliated to Yangzhou University, Yangzhou, China; ^5^ Department of Neurology, Yizheng People's Hospital, Yangzhou, China; ^6^ Department of Ultrasound, Guangdong Second Provincial General Hospital, School of Medicine, Jinan University, Guangzhou, China; ^7^ Department of Neurology, Jiangdu People's Hospital affiliated to Yangzhou University, Yangzhou, China

**Keywords:** immune checkpoint inhibitors, Sintilimab, PD-1, immune-related adverse events, inflammatory myopathy

## Abstract

The use of immune checkpoint inhibitors (ICIs) has shown remarkable efficacy in the treatment of various malignancies, significantly reshaping cancer treatment. However, as a result of the widespread use of ICIs, several immune-related adverse events (iRAEs) have emerged, some of which can be rare and potentially fatal. In this paper, we reported the earliest case of Sintilimab used in the treatment of esophageal cancer with severe inflammatory myopathy (involving the cardiac, respiratory, and skeletal muscles)in China. This patient was an elderly female who presented to our institution with progressive limb weakness and ptosis. Prior to the onset of symptoms, the patient had undergone a radical esophagectomy for esophageal cancer, experienced several cycles of of radiotherapy and chemotherapy, as well as two doses of Sintilimab treatment. Shortly after initiating immunotherapy, the patient developed symptoms including bilateral ptosis, limb weakness, and difficulty swallowing and breathing. The levels of creatine kinase and troponin I in the patient’s blood were significantly elevated, and positive results were observed for anti-skeletal and anti-cardiac muscle antibodies, indicating that the patient might be developing ICIs-related inflammatory myopathy. Fortunately, the patient responded well to treatment including corticosteroids, plasmapheresis, intravenous immunoglobulin, and other supportive therapies. Here, we discuss the incidence, mechanisms, and management strategies of fatal iRAEs. Early detection and timely intervention may be critical in reducing the incidence and mortality rates of iRAEs and improving patient outcomes.

## Introduction

Immune checkpoint inhibitors (ICIs) have become a revolutionary treatment modality across a variety of refractory malignancies. By blocking checkpoint proteins like PD-1 and CTLA-4, ICI drugs remove the barriers that prevent the immune system from attacking cancer cells (1). Sintilimab was first approved by the China National Medical Products Administration in December 2018, marking its efficacy in treating relapsed or refractory classical Hodgkin lymphoma after failure of at least two lines of systemic chemotherapy (2). So far, Sintilimab has also been approved for the treatment of various other solid cancers, including non-small cell lung cancer, hepatocellular carcinoma, and melanoma, and there were few reports on esophageal cancer (3, 4).. As Sintilimab becomes more widely used, an increasing number of immune-related adverse events (iRAEs) have been identified. While most of these iRAEs are mild and can be managed with supportive care, a few serious conditions such as respiratory failure and myocarditis may prove fatal if not diagnosed and treated promptly. In this paper, we presented the earliest rare case in China of severe inflammatory myopathy (simultaneously involving the heart, respiratory and skeletal muscles) in the treatment of esophageal cancer with Sintilimab, highlighting the importance of early identification and intervention for this potentially fatal immune-related complication.

## Case presentation

An elderly woman with a 30-year history of type 2 diabetes was referred to our institution on late December 2021, with a diagnosis of progressive limb weakness and ptosis. She had previously undergone radical resection for esophageal cancer. The patient received relevant radiotherapy and immunotherapy in our institution on mid-November.

**Figure 1:**
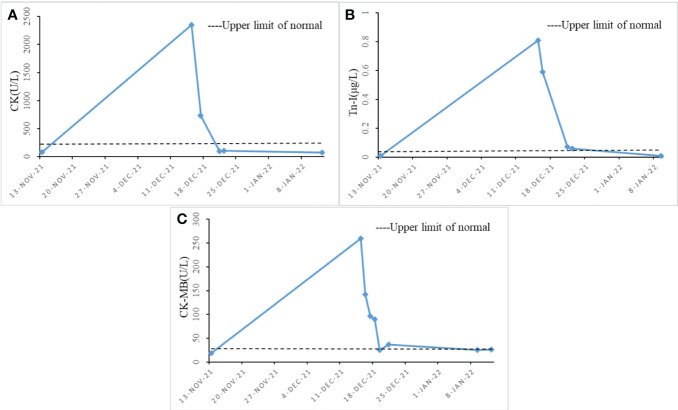
Changes in cardiac function index levels during hospitalization (x-axis = date, y-axis = unit symbol). **(A)** shows the level change of CK; **(B)** shows the level change of Tn-I. **(C)** shows the level change of CK-MB.

The patient underwent radical resection for esophageal cancer at our institution on mid-July 2020. Multiple rounds of chemotherapy and radiotherapy were performed from late August to mid-December. After discharge, the patient received regular follow-up and related examinations, and the tumor did not progress significantly. On mid-November 2021, the patient came to our institution again due to unexplained hoarseness. During laryngoscopy, no signs of tumor were found in the nasopharynx. The vocal fold movement was also normal. As a result, the patient was asked to undergo a further detailed examination. After some tests, the glycoconjugate antigen CA72-4 revealed 35.52 U/mL, and CT scans of the neck and chest showed postoperative changes of oesophageal cancer and a suspicious soft tissue focus in the left posterior trachea. Based on the combination of the tumour markers and CT scans, it was suggested that there was a high probability of local recurrence. Palliative radiotherapy was administered to the patient on mid-November, targeting the recurrent foci. The prescribed dose was 200 cGy/F for PTV and 5000 cGy/25 F for DT. Due to concerns about the patient’s ability to tolerate systemic chemotherapy, with the consent of the patient and his family, the patient was given immunotherapy with Sintilimab 200mg in mid-November and early December. However, on early December, the patient began showing concerning symptoms. The patient experienced difficulty opening both eyes and swallowing, and also had some minor weakness in their limbs. Fortunately, she was still able to move around at this point, but her condition would need to be closely monitored moving forward. On mid-December 2021, the patient underwent blood tests to measure their creatine kinase(CK) levels, which were found to be extremely high at 2351 U/L. The patient also had a creatine kinase-MB(CK-MB) level of 260 U/L and a troponin-I(Tn-I) level of 0.81 μg/L. To further investigate the matter, a bedside electrocardiogram (ECG) was performed immediately. The ECG revealed no significant ST-segment elevation and appeared to be normal. However, the results suggested the possibility of immune myocarditis, prompting physicians to administer empirical treatment with methylprednisolone. Patients were treated with methylprednisolone (2 mg per kilogram of body weight per day intravenously for 5 days, then 4mg per day orally for 2 weeks). During the patient’s hospitalization, CK, Tn-I, and CK-MB markers were regularly monitored. As seen in **Figure 1**, methylprednisolone treatment effectively reduced the levels of all three markers.

**Figure 2:**
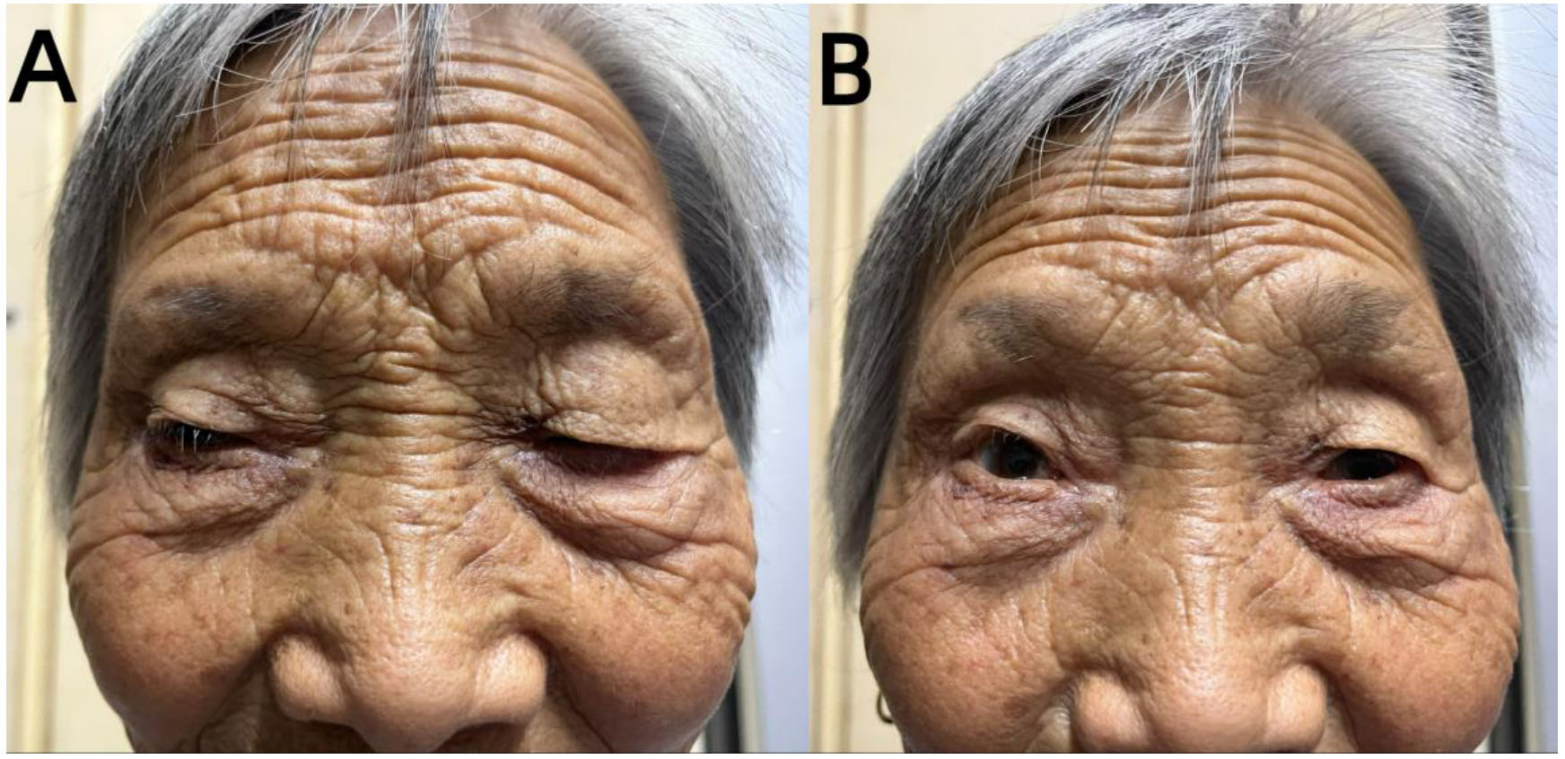
Paralysis/myasthenia gravis: an elderly female with eye movement disorder and ptosis. The symptoms of ptosis were compared between pre-intervention **(A)** and post-intervetion **(B)**.

**Figure 3:**
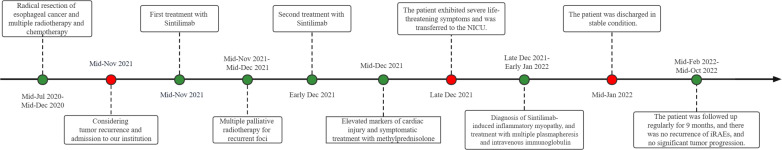
Timeline of disease onset, progression, diagnosis, treatment, and follow-up.

Unfortunately, the patient exhibited a host of concerning symptoms, including significant limb weakness, dysphagia, hypercapnia, respiratory failure, ptosis, and ocular muscle paralysis on late December which prompted her to be transferred to the Neuro-Intensive Care Unit (NICU) for further treatment. The neurological examination found that the patient was lethargic, dyspnea, bilateral ptosis, difficulty opening eyes, bilateral pupils were large and round, sensitive to light reflex, weakness in raising the head and turning the neck, muscle strength of upper limbs was grade 3, muscle strength of lower limbs was grade 2, muscle tension of limbs was low, tendon reflexes (-), pathological signs were negative. Symptomatic treatment, such as tracheal intubation, antibiotic therapy, and nutritional support, was administered in a timely manner where appropriate. Routine blood test showed that the patient’s white blood cell (WBC) and C-reactive protein (CRP) levels were significantly elevated, which indicated that there may be a severe inflammatory reaction. In addition, immune-related antibodies were detected and the results could be found in **Table 1**. Different antibodies were detected by different methods, including enzyme-linked immunosorbent assay (ELISA), Western blotting (BLOT), LINEBIOT and indirect immunofluorescence test (IIFT). Anti-skeletal muscle and myocardial antibodies were tested using the “Autoantibody Spectrum IgG Test Kit” produced by EUROIMMUN, Germany, product number FA1800-6, and in strict accordance with the instructions for use.The patient tested positive for anti-skeletal muscle and anti-cardiac muscle antibodies alongside the aforementioned elevated levels of CA72-4, CK, Tn-I, and CK-MB (referred to in the appeal). An array of relevant tests were performed on the patient to eliminate the possibility of an underlying primary Guillain Barre syndrome or myasthenia gravis, including repetitive electrical nerve stimulation tests, neostigmine tests, electromyography, and cerebrospinal fluid tests. Repetitive electrical nerve stimulation test and neostigmine test were negative, while electromyography showed myogenic damage.The results of the cerebrospinal fluid tests can be found in **Table 2**. To minimize potential errors from a single test, the patient underwent additional lumbar puncture examinations on late December, both of which failed to demonstrate the typical protein-cell dissociation. The first cerebrospinal fluid test demonstrated high glucose levels, which were likely due to the patient’s chronic poor glycemic control. Conversely, the second test revealed the presence of elevated antibody levels, possibly caused by intravenous immunoglobulin treatment. Considering the patient’s clinical presentation and test results collectively, it was concluded that the patient may be experiencing a diagnosis of ICI-related inflammatory myopathy. The patient underwent three plasmapheresis on late December. After the second plasmapheresis, the patient experienced a significant improvement in both breathing and eye-opening symptoms. Subsequently, the patient received intravenous immunoglobulin treatment (400mg per kilogram of body weight per day for five days) from late December to early January. Plasmapheresis was performed prior to immunoglobulin treatment to prevent the loss of effectiveness of the immunoglobulins. Following the stabilization of the patient’s condition, he was transferred from the NICU to the general ward on late December. The patient was discharged on mid-January 2022, after a 2-week period of observation and symptomatic treatment. As indicated in **Figure 2**, the patient was free from symptoms of ptosis at the time of discharge. The patient was followed up regularly for 9 months. During this period, there was no recurrence of immune-related adverse events (iRAEs), and the tumor did not progress significantly. **Figure 3** illustrates the primary time nodes of patients’ disease occurrence, development, diagnosis, treatment, and follow-up.

**Table 1:** Laboratory data on immune-related antibodies in serologic examination.

Antibody Type	Detection Method	Test result
ANA	IIFT	Nucleolar 1:160
Anti-RNP Ab	ELISA	Negative
Anti-Sm Ab	ELISA	Negative
Anti-Ro52 Ab	ELISA	Negative
Anti-Jo-1 Ab	LINEBIOT	Negative
Anti-SSA Ab	ELISA	Negative
Anti-SSB Ab	ELISA	Negative
Anti-Scl-70 Ab	ELISA	Negative
Anti-PM-Scl Ab	LINEBIOT	Negative
Anti-PCNA Ab	ELISA	Negative
Anti-Mi-2 Ab	LINEBIOT	Negative
Anti-SRP Ab	LINEBIOT	Negative
Anti-PL-7 Ab	LINEBIOT	Negative
Anti-NMDA Receptor Ab	IIFT	Negative
Anti-VGKC Complex Ab	IIFT	Negative
Anti-Hu Ab	BLOT	Negative
Anti-Yo Ab	BLOT	Negative
Anti-AchR Ab	ELISA	Negative
Anti-RyR Ab	ELISA	Negative
Anti-LRP4 Ab	ELISA	Negative
Anti-Titin Ab	BLOT	Negative
Anti-SOX-1 Ab	BLOT	Negative
Anti-Musk Ab	IIFT	Negative
Anti-skeletal muscle Ab	IIFT	1:320
Anti-myocardial Ab	IIFT	1:320

ANA, Anti-nuclear antibody; IIFT, Indirect Immunofluorescence Test; ELISA, Enzyme-linked immunosorbent assay; RNP, Ribonucleoprotein; Ab, Antibody; Sm, Smith; BLOT, Western blotting; SSA, Sjögren’s-syndrome-related antigen A; SSB, Sjögren’s-syndrome-related antigen B; Scl, Scleroderma; PM-Scl, Polymyositis-scleroderma; PCNA, Proliferating cell nuclear antigen; SRP, Signal recognition particle; NMDA, N-Methyl-D-Aspartate; VGKC, Voltage-Gated Potassium Channel; AchR, Acetylcholine receptor; RyR, Ryanodine receptor; LRP4, Low-density lipoprotein receptor-related protein 4; SOX-1, Sex-determining region Y-Box protein 1; Musk, Muscle-specific kinase.

**Table 2:** Laboratory data from cerebrospinal fluid examination.

At hospitalization			
	Mid-late Dec 2021	Late Dec 2021	
Test items	Detection value	Detection value	Normal value
Appearance	colorless	colorless	colorless
Pressure	71	110	70-180(mmH_2_O)
Nucleated cell	0.007	0.002	0-0.008(10^9/L)
WBC	0.004	0.001	0-0.005(10^9/L)
RBC	0	0	0 (10^9/L)
Protein	383.4	248.2	150-450(mg/L)
Glu	13.16	4.29	2.5-4.5(mmol/L)
Cl	148.2	121	120-132(mmol/L)
LAC	1.70	1.23	1.19-2.09(mmol/L)
LDH	37	22	10-45(U/L)
IgG	12.1	24.20	10.00-40.00(mg/L)
IgM	3.22	6.94	0.00-13.00(mg/L)
IgA	1.07	5.00	0.00-6.00 (mg/L)
CN	Negative	Negative	Negative
Bacterial culture	Negative	Negative	Negative
Pandy’s Test	Negative	Negative	Negative

Dec, December; WBC, white blood cells; RBC, red blood cells; Glu, glucose; Cl, chloride; LAC, Lactate; LDH, lactate dehydrogenase; Ig, immune globulin; CN, cryptococcus neoformans.

## Discussion

Immunotherapy is a promising and emerging therapeutic approach for malignancies. However, the widespread use of immune checkpoint inhibitors (ICIs) has led to the emergence of an increasing number of immune-related adverse events (iRAEs). Previous studies have shown that severe iRAEs occur in 15% of all patients with iRAEs, with pneumonia and pulmonary infection being the most commonly reported (5).The study by Moreira et al. found that the incidence of concomitant neuromuscular side effects in patients treated with ICIs was only about 1% (6). Although neuromuscular-related high-grade iRAEs are uncommon in ICIs, the risk of patient mortality through heart failure, arrhythmias, or complications from a protracted stay in the ICU is substantial. Higher morbidity and mortality from myocarditis may be associated with the combination of ICIs, which involves multiple target organs, leading to the development of multiple diseases (7). Some cancer patients have developed myocarditis after receiving only 1-2 anti-PD-1 treatments, with 25% of these cases overlapping with myocarditis or MG in the presence of excitatory autoantibodies (8). In this case, the patient’s experience was remarkably like to the patient’s detailed in the appeal article. The patient developed ICIs-induced myocarditis on the seventh day after receiving the second cycle of sintilimab, which is consistent with the typical timeframe for myocarditis onset (9). This outcome supports the conclusion that ICIs-induced myocarditis is more likely to occur after two to three cycles of the medication.While the endomyocardial biopsy is considered the “gold standard” for diagnosing myocarditis, it is rarely performed due to its invasiveness and associated risks such as perforation (10). Therefore, cardiac magnetic resonance (CMR) imaging is the preferred diagnostic tool for myocarditis due to its noninvasiveness and ease of use (10). Unfortunately, the patient’s family declined the examination due to financial constraints. Although elevated troponin levels are not specific to any particular etiology, they are the most sensitive marker of myocardial injury, and anticardiac antibodies were also positive. Empirical treatment with glucocorticoids resulted in favorable clinical outcomes for the patient. The patient exhibited elevated creatine phosphokinase levels and tested positive for anti-skeletal muscle antibodies. The patient’s repetitive nerve electrical stimulation test and neostigmine test were both negative; and the patient’s limb skeletal muscles did not show fluctuating weakness and fatigability, nor did they have the performance of “light in the morning and heavy in the evening”. These test results and clinical manifestations suggested that this patient might also have immune-related myositis rather than myasthenia gravis. Myositis and myocarditis induced by PD-1 inhibitors may have a common pathophysiological mechanism, but the cellular targets they attack are different. Current understanding posits PD-1-induced myopathy as a potential independent subtype of inflammatory myopathy; however, further research is required to elucidate its precise mechanism and classification.

Currently, there is no standardized diagnosis for iRAEs caused by ICIs, and diagnosis is primarily based on experience with autoimmune neurological diseases (11). Its pathogenesis has not yet been fully elucidated. According to some authors, tumor cells and nerve and muscle tissues may share the same antigenic determinants. These determinants are typically expressed on muscle cells but are expressed abnormally on tumor cells (12). As a result, although ICIs enhance the immune attack on tumors, they may also cause secondary immune damage to the muscular system (13). From a pathophysiological perspective, PD-1 inhibitors may increase the baseline T cell immune response to the tumor. T cell immunity is the primary target of tumor immunotherapy, and it can recognize and eliminate antigen-expressing cells through CD8+ cytotoxic T cells (14). The main cause of iRAEs, however, may be an autoimmune reaction brought on by an augmented immune response fueled by T-cell activation. Moreover, some authors suggest that ICIs treatment could exacerbate nervous system paraneoplastic syndrome (PNS) and trigger iRAEs. Vogrig et al. demonstrated that treatment with ICIs increased the autoimmune response in Ma2 antibody-positive patients, resulting in the development of immune-related encephalitis (15). This suggested that patients with autoantibodies may face a significantly elevated risk of developing immune-related adverse events (iRAEs) after receiving immune checkpoint inhibitors (ICIs) treatment. However, further research is necessary to precisely elucidate the relationship between these variables.

There is currently no consensus or unified clinical guidelines for the treatment and management of iRAEs caused by ICIs. Treatment decisions primarily rely on clinical judgment and experience. According to the American Society of Clinical Oncology (ASCO) guidelines, for grade 1-2 iRAEs, immunotherapy can be suspended and low-dose corticosteroids can be given (16). Grade 3 iRAEs usually require the suspension of immunotherapy and the initiation of high-dose corticosteroids, which should be gradually tapered over at least 4-6 weeks. Refractory cases might require additional immunosuppressive therapy, such as intravenous immunoglobulin or plasma exchange. For grade 4 iRAEs, immunotherapy should be permanently discontinued, and specific treatment should be given based on the different symptoms (16). In our report, the patient experienced severe grade 3-4 iRAEs. Shortly after the second cycle of Sintilimab treatment, the patient developed severe myocarditis, which was managed with methylprednisolone to aid in the patient’s survival during the acute phase of cardiotoxicity. It was later discovered that the patient was also experiencing other conditions, such as myositis and respiratory failure, which were managed with timely respiratory support, plasmapheresis, and intravenous immunoglobulin, among other symptomatic treatments. Previous studies have reported that approximately 42% of patients with ICIs-related myocarditis may be accompanied by other serious iRAEs, with myositis and myasthenia gravis being the most common (8). A separate study discovered that patients with comorbid myasthenia gravis and myocarditis or myositis exhibited elevated mortality rates compared to individuals with myasthenia gravis without these conditions (17). The results indicated that the concurrent occurrence of multiple severe iRAEs could pose a considerable challenge for clinicians and lead to decreased survival rates among patients. Furthermore, around 50% of these patients may experience respiratory failure, which is a critical condition necessitating timely and precise symptom recognition, along with suitable ventilatory support (18).. The patient in this report was fortunate to have received timely and effective management of their iRAEs.

In conclusion, during the treatment of patients with ICIs, it is imperative to consistently monitor for the presence of iRAEs. Thorough comprehension of patients’ noteworthy clinical features, comprehensive utilization of test results, and prompt diagnosis are of utmost importance.If immune-related adverse events occur, the severity should be promptly assessed, and ICIs should be discontinued. Corticosteroids, plasmapheresis, or intravenous immunoglobulin should be immediately used as appropriate. Regularly following up to observe whether symptoms worsen, recur, or the primary tumor progresses is essential to optimize the disease prognosis.

## Conclusion

Sintilimab have emerged as a prevalent therapeutic approach for diverse cancer types. The present case report demonstrates that sintilimab-induced inflammatory myopathy represent a rare yet potentially lethal adverse event. Timely identification and intervention are crucial in reducing the occurrence and mortality of immune-related adverse events (iRAEs), consequently improving patient prognosis.

## Data availability statement

The original contributions presented in the study are included in the article/supplementary material. Further inquiries can be directed to the corresponding author.

## Ethics statement

Written informed consent was obtained from the individual(s) for the publication of any potentially identifiable images or data included in this article. Written informed consent was obtained from the participant/patient(s) for the publication of this case report.

## Author contributions

GH: Conceptualization, Methodology, Software, Visualization, Writing – original draft, Writing – review & editing. YY: Data curation, Methodology, Software, Supervision, Writing – review & editing. HZ: Data curation, Investigation, Methodology, Supervision, Writing – review & editing. HS: Software, Supervision, Validation, Visualization, Methodology, Conceptualization, Data curation, Writing – review & editing. ZZ: Methodology, Software, Supervision, Conceptualization, Writing – review & editing. JY: Methodology, Software, Supervision, Visualization, Conceptualization, Data curation, Writing – review & editing. LZ: Conceptualization, Data curation, Methodology, Software, Supervision, Validation, Writing – review & editing.
